# Consumer Preference Analysis on Attributes of Milk Tea: A Conjoint Analysis Approach

**DOI:** 10.3390/foods10061382

**Published:** 2021-06-15

**Authors:** Ardvin Kester S. Ong, Yogi Tri Prasetyo, Ma. Althea Deniella C. Libiran, Yuanne Mae A. Lontoc, Joyce Anne V. Lunaria, Adelaine M. Manalo, Bobby Ardiansyah Miraja, Michael Nayat Young, Thanatorn Chuenyindee, Satria Fadil Persada, Anak Agung Ngurah Perwira Redi

**Affiliations:** 1 School of Industrial Engineering and Engineering Management, Mapúa University, 658 Muralla St., Intramuros, Manila 1002, Philippines; aksong@mapua.edu.ph (A.K.S.O.); mnyoung@mapua.edu.ph (M.N.Y.); thanatorn@webmail.npnu.ac.th (T.C.); 2 School of Graduate Studies, Mapúa University, 658 Muralla St., Intramuros, Manila 1002, Philippines; 3 Young Innovators Research Center, Mapúa University, 658 Muralla St., Intramuros, Manila 1002, Philippines; madclibiran@mymail.mapua.edu.ph (M.A.D.C.L.); ymalontoc@mymail.mapua.edu.ph (Y.M.A.L.); javlunaria@mymail.mapua.edu.ph (J.A.V.L.); amanalo@mymail.mapua.edu.ph (A.M.M.); 4 Department of Business Management, Institut Teknologi Sepuluh November, Kampus ITS Sukolilo, Surabaya 60111, Indonesia; bobard.m@outlook.com (B.A.M.); satriafp@gmail.com (S.F.P.); 5 Logistics and Supply Chain Management Program, Nakhon Pathom Rajabhat University, Nakhon Pathom 73000, Thailand; 6 Industrial Engineering Department, BINUS Graduate Program-Master of Industrial Engineering, Bina Nusantara University, Jakarta 11480, Indonesia; wira.redi@binus.edu

**Keywords:** milk tea, conjoint analysis, tapioca pearls, market analysis

## Abstract

Milk tea is a famous drink that has been heavily consumed since 2011. This study aimed to determine the combination of milk tea attributes that were most preferred using a Conjoint Analysis Approach. Specifically, this study utilized different attributes such as the size of tapioca pearls, sugar level, price range, brands, type of milk tea, cream cheese inclusion, and the amount of ice. Conjoint analysis with the orthogonal design was utilized to evaluate the preference of milk tea among consumers. The results showed that pearl size was the attribute most considered by consumers (29.137%), followed by sugar level (17.373%), the amount of ice (17.190%), the type of drink (13.421%), price (11.207%), and the least considered were cream cheese inclusion (9.525%) and the brands (2.147%). The findings of this study will be beneficial to milk tea firms about consumer preferences regarding the various attributes of milk tea. Finally, the result of this study could be applicable to different beverage-focused studies worldwide.

## 1. Introduction

Milk tea is a famous tea-based drink that originated in Tainan and Taichung, Taiwan, in the 1980s [1]. It was invented by a Taiwanese tea shop owner, Liu Han-Chieh, and his product development manager, Lin Hsiu Hui [2]. The milk tea that we know today was discovered by adding different ingredients such as fruits, syrups, and tapioca pearls into different tea with milk beverages [2].

Milk tea first gained popularity in the 1990s throughout Asia and became more popular in the United States and Europe in the 2000s [3]. The popularity turned into a global phenomenon due to the versatility and flexibility of the toppings and flavor combinations that consumers could choose from [4]. With the global phenomenon of milk tea, it eventually hit the market with a $2.4 billion value in 2019 and is estimated to reach $4.3 billion by 2027 [1].

In the same article [1], the projection of the increase in the market will be in North America followed by Asia-Pacific, Europe, and LAMEA. For North America, it is said that the U.S. will be at the top followed by Canada and Mexico. For Europe, places like United Kingdom, Germany, France, and Italy will be the top regions. Moreover, Asia-Pacific has China as the top country followed by Japan, India, Australia, and Taiwan. LAMEA will cover Brazil, Saudi Arabia, South Africa, and Turkey [1].

Taking into consideration the components of milk tea, its sweet and original flavor was its selling point in different regions [1]. With its sweet flavor, it captured the hearts of people and eventually entered the beverage market [5]. Milk tea shops eventually spread and are located throughout malls, parks, and neighborhoods [6]. With that, the significant spread of milk tea shops has been observed around the world. Shops coming from different Asian regions like the Chatime group, CoCo, GongCha, all of which are from China and Taiwan, and Tiger Sugar from Korea, spread [7]. These brands eventually went international and people in those countries capitalized on the trend and made their own brands. Fortune Business Insight [7] included the U.S. brand Boba Loca Inc., Lollicup USA, Inc., and Kung Fu Tea. Moreover, United Kingdom has their own brand called Happy Lemon.

**Figure 1:**
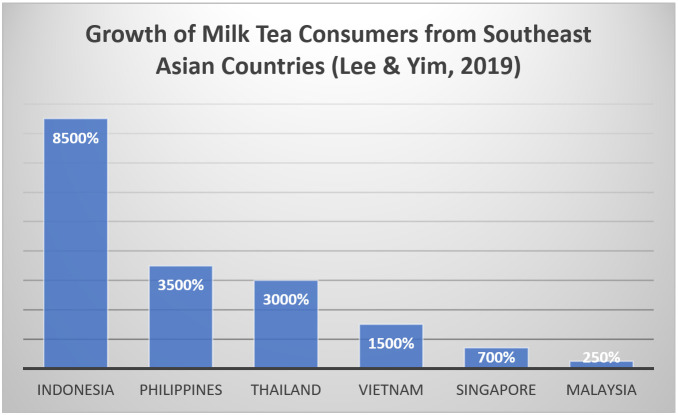
Percentage growth of milk tea consumers from Southeast Asian Countries. Adapted from [9].

The different brands of milk tea became a huge option among consumers [8]. Consunji and Capili [8] found that consumers would prefer milk tea for its affordability compared to other popular beverages such as coffee. For that reason, milk tea became the largest beverage trend starting in 2011. In Asia, milk tea’s popularity significantly increased in 2018 with a 3500% growth, as indicated in Figure 1 [9]. Lee and Yim [9] then revealed that the Philippines and Thailand ranked second after a huge difference from Indonesia in Southeast Asia with the largest number of milk tea consumers. This implies that several milk teas brands, either local or international, are becoming more popular.

Among the brands that are popular and are first in the market are CoCo, GongCha, Chatime, Macao Imperial, and Tiger Sugar [1]. CoCo has been operating since 1977, with over 4000 stores worldwide [10]. With over 1500 stores, GongCha has been producing quality products with a wide range of customized milk tea flavors [11]. On the other hand, Chatime, known for their pearl milk tea, opened their first branch in the Philippines in 2011 [12] and eventually grew internationally. Recently, Macao Imperial and Tiger Sugar have been competing with other leading brands. Consumers have been buying these brands because of their distinct milk tea taste and attributes.

The different milk tea brands have their own specialty; however, they offer different levels of attributes. These attributes may include brands, price range, size of tapioca pearls, sugar level, the amount of ice, cream cheese inclusion, and the type of milk tea. Milk tea attributes are consumers’ preferred set of combinations on their ordered milk tea. For instance, when someone buys a milk tea, he or she is being asked about the type of milk tea, sugar level, size of the pearls, and level of ice. Thus, it is very important to analyze these attributes, particularly for the marketing strategy.

There were previous studies relating to milk tea and the utilization of conjoint analysis on other beverages. Shih et al. [13] conducted a study that aimed to investigate the consumers’ beverage purchase behavior and their preference for different beverage groups in Tainan, Taiwan, by using Descriptive Statistics and Pearson Correlation Coefficients. It showed that there is a positive correlation between the product attribute and consumer purchase decision [13]. Similarly, Khanna [14] utilized conjoint analysis to understand the factors impacting consumer preferences and their evolving purchase intention for numerous products available in highly dynamic beverage industries. The results showed that branding, health benefits, price, and calories are the various factors that determine the major change in the focus of the consumers toward milk and fruit-based drinks [14]. Moreover, Lee and Vega [15] determined the factors affecting consumers’ purchasing behavior of selected milk tea shops to sustainability. It was concluded that the management of milk tea stores should focus on providing healthy alternatives and innovate exceptional tea products that are affordable yet are high quality to achieve full customer satisfaction and loyalty.

Regardless of the availability of studies about milk tea, there is scarce literature focusing on the milk tea preferences. In the Philippines, De Jesus [16] only focused on the milk tea industry in the areas of Nueva Ecija, Philippines. The results showed that milk tea shop owners are prioritizing convenience through fast service, quality ingredients, and by fostering loyal customers in their businesses [16]. In addition, Supranes and Van [17] did a study focusing on the “Milk Tea War” using ARIMA models to evaluate the competition among six popular milk tea brands in the Philippines. The results from the ARIMA models hinted the brands that have gained momentum in 2019 are the preferred brands [17]. Given the different works of literature, there were no studies that dealt with consumer preferences on milk tea using a Conjoint Analysis Approach. Thus, it would be effective to use Conjoint Analysis approach for analyzing the consumer preferences specifically in terms of milk tea.

Conjoint analysis is a research tool widely used in marketing and consumer research [18,19,20,21,22,23]. In this method, the respondents are presented with various combinations of components formed by the classified attributes and levels of a product or service [24,25,26,27]. The respondents’ preferences are then assessed in the form of ratings, rankings, or choices for those hypothetical products or services [28]. With this technique, entrepreneurs can recognize the impacts of each attribute on consumers’ purchase intentions [29,30,31,32]. Understanding the consumers’ purchase intention is important for businesses to successfully develop, compete, and market their products.

This study aimed to determine the combination of milk tea attributes that were most preferred by the consumers using a Conjoint Analysis Approach. Specifically, this study utilized different attributes such as brands, price range, size of tapioca pearls, sugar level, amount of ice, inclusion of cream cheese, and type of milk tea. Conjoint analysis with the orthogonal design was utilized to evaluate the preference of milk tea among consumers. The findings of this study will be beneficial to milk tea firms about consumer preferences regarding the various attributes of a milk tea. Moreover, the result of this study could be applicable to different beverage-focused in other countries.

## 2. Methodology

### 2.1. Participants

The study utilized random sampling to gather respondents through the dissemination of an online survey [33]. Sethuraman et al. [34] suggested that online distribution of surveys was plausible when doing conjoint analysis. The survey was accessible from 12 February 2021 to 24 February 2021. There were a total of 1061 Filipino respondents who participated in answering the 34 combined attributes about milk tea preference.

### 2.2. Demographics

Table 1 presents the demographics of the study. Among the 1061 Filipino respondents, 25.8% were male, 73.3% were female, and 0.8% were unspecified. Most of the respondents were aged 15–24 years old (84.2%). The other respondents were aged below 15 years old (4.2%), 25–34 years old (4.8%), 35–44 years old (3.7%), 45–54 years old (2.2%), and above 54 years old (0.6%). Around (84%) of the respondents had a monthly allowance of less than 15,000 Php. The monthly allowance of other respondents was 15,000–30,000 Php (10.6%), 30,001–45,000 Php (2.5%), 45,001–60,000 Php (2.5%), 60,001–75,000 Php (0.7%), and above 75,000 Php (1.1%). Approximately 69.7% of the respondent drink milk tea once a week. Most of the respondents were located at NCR (36.8%), Region III (29.3%), and Region IV-A (21.5%).

### 2.3. Conjoint Design

Table 2 presents the attributes of milk tea. This study considered pearl size (big, small, or no pearls), sugar level (more, normal, less, or no sugar), price (120 PhP (2.46 USD), 150 PhP (3.08 USD), or 180 PhP (3.70 USD)), Brand (CoCo, Macao Imperial, GongCha, Tiger Sugar, or Chatime), Type (Milk tea or Fruit Tea), cream cheese inclusion, and amount of ice (more, normal, less, or no ice). A total of 7 attributes were considered in this study.

The first attribute, pearl size, refers to the sinkers that can or cannot be present in the milk tea. There are plenty of choices for toppings, but the pearls seem to be the unbeatable and original go-to topping for most milk tea drinks [9,35]. Pearls are traditional milk tea add-ons known for their chewy and squishy texture as well as their sweet taste [35]. For the pearl size, three levels were considered: big pearls, small pearls, and no pearls. Big pearls refer to the standard size of tapioca pearls, while the small pearls are mini version and about half the size of the bigger one, since smaller pearls may be easier to chew than the larger ones [36,37]. These levels reflect the usual offerings of the local milk tea brands.

Second, sugar level has a major influence on consumers’ preference on the sweetness level of milk tea. The percentage of sugar changes depending on the preferred sweetness of the customer [38]. Some milk tea consumers prefer their milk tea not too sweet in order to control sugar intake. With the idea of milk tea customization, all consumers are given an option to choose the level of sugar content in their drink. The consumer can opt for 75%, 50%, 35%, or even lower percentages of sugar [39]. For sugar level, four levels were considered: more sugar, normal sugar, less sugar, and no sugar. These levels are usually the sugar level variations of milk tea brands, either local or international.

Third, price is one of the key factors that affects the consumers’ preference on how much money they are willing to spend on milk tea. Consumers’ price consciousness has a positive influence on their purchase intention towards bubble milk tea [40]. The range of prices depends on the components present in the milk tea. For the price, three levels were specified: 120 PhP (2.46 USD), 150 PhP (3.08 USD), and 180 PhP (3.70 USD). These levels are the typical price range of the locally available milk teas.

**Figure 2:**
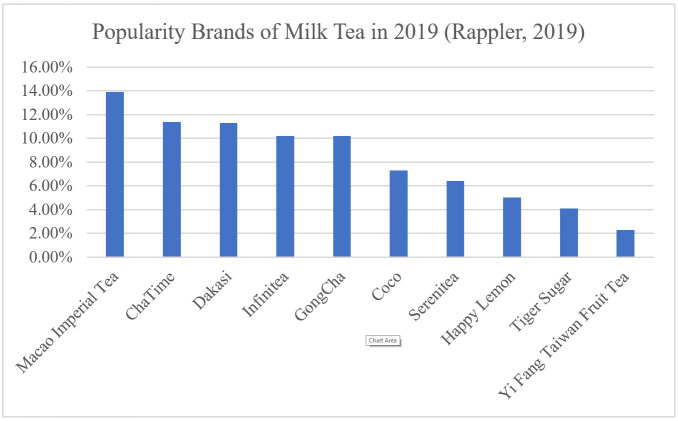
Milk tea popularity in the Philippines. Adapted from [43].

Fourth, brand serves as a way for consumers to recognize the products of a manufacturer [41]. The study by Wen and Aun [40] revealed that brand has a significant influence on consumers’ purchasing intentions towards milk tea. With this, five different levels were identified in terms of brands: CoCo, Macao Imperial, GongCha, Tiger Sugar, and Chatime. These are the selected levels since these milk tea brands are popular [1,42]. Moreover, these levels are among the most mentioned milk tea brands on Philippine Twitter in 2019. According to Rappler [43], the popularity of these brands was as follow (Figure 2): Macao Imperial (13.9%), Chatime (11.4%), GongCha (10.2%), CoCo (7.3%), and Tiger Sugar (4.1%).

Fifth, the type, which depends on how the beverage is mixed with the tea base of the milk tea, was also included in the considered attributes. In this attribute, two levels were considered: milk tea and fruit tea. In regular milk tea, the tea base is often shaken with milk, powdered milk, condensed milk, or non-dairy creamer. On the other hand, fruit teas are usually the slushy versions of milk tea which are infused with fruit juices [44]. Despite the higher tendency of Filipinos to have a sweet tooth [45], Saalia et al. [46] suggested that non-dairy components can also benefit some consumers who are lactose intolerant, as well as those who are looking for a healthy alternative to dairy milk. Therefore, this attribute is also seen as a significant factor affecting consumer preferences.

Sixth, cream cheese inclusion also influences the decision of consumers when buying a milk tea. Cream cheese is a type of soft cheese that has high protein and low fat and is used as an ingredient in many food applications [47]. Due to the health benefits of cream cheese, cream cheese inclusion in milk tea is becoming a trend. For cream cheese inclusion, two levels were considered: with cream cheese and without cream cheese. These are the levels considered on cream cheese inclusion since cream cheese is an optional ingredient in milk tea.

Lastly, the amount of ice is also one of the attributes considered as milk tea is usually served cold [15]. This can also be customized by the consumers in order to suit their preferences. Given this, the attribute has four levels: more ice, normal ice, less ice, and no ice. These variations were considered due to the awareness of some consumers that the amount of ice put on drinks affects the ratio between the water and the actual beverage [48]. Thus, this attribute also contributes to the factors influencing the consumers’ decision when buying milk tea.

### 2.4. Statistical Analysis

The conjoint analysis with the orthogonal design utilized SPSS 25. A total of 34 stimuli were generated by the SPSS. The orthogonal design was utilized to ensure the reasonable number of stimuli that were evaluated by the participants. Table 3 presents the 34 stimuli evaluated by a 7-point Likert scale ranging from 1 as “strongly disagree” to 7 as “strongly agree”.

## 3. Results

Table 4 and Table 5 represent the utilities and the average importance score of preference of milk tea. Based on the average importance scores, pearl size was the most important attribute for consumers, followed by sugar level, amount of ice, type, price, cream cheese inclusion, and brand. To determine the utilities allocated to each level of the attribute, Table 4 presents the utility scores obtained by each attribute. First, with the pearl size attribute, consumers preferred big pearls rather than small pearls since it had the highest utility score. Second, for the sugar level attribute, normal sugar was most desired by the consumers, followed by more sugar. Third, within the ice attribute, the normal ice was the most preferred of the consumers followed by more ice. Fourth, in the type of attribute, milk tea obtained the highest utility score. Fifth, under the price attribute, the lower the price, the higher the utility score. Sixth, within the cream cheese inclusion, cream cheese preference was the most favored. Lastly, under the brand attribute, GongCha obtained the highest utility score, followed by Macao Imperial, CoCo, and Chatime.

Table 6 represents the ranking of the 34 stimuli. It was seen that among the 34 stimuli, combination 19 ranked first as it was the most preferred of the consumers. The attributes under the combination 19 were big pearls, normal sugar, 120 PhP (2.46 USD), Macao Imperial, milk tea, with cream cheese, and more ice. On the other hand, it could be seen that combination 13 which consists of no pearls, more sugar, 150 PhP (3.08 USD), Chatime, fruit tea, with cream cheese, and no ice ranked the last as it was the least favored by the consumers.

Table 7 represents the correlation of the stimulus created in this paper. The value of Pearson’s R is 0.966 and the value of Kendall’s Tau is 0.861. As the obtained values are close to 1, these show a strong relationship between the observed and estimated preferences [23]. In addition, this study added two holdouts to determine the consistency among the responses. With that, the Kendall’s coefficient for holdouts has a value of 1.000, which implies the high quality of the collected data.

## 4. Discussion

Among the different stimuli, the conjoint analysis revealed that the most favored stimulus by milk tea consumers was big pearls, normal sugar, 120 PhP (2.46 USD), GongCha, milk tea, and normal ice with a total utility score of 1.297. The least preferred stimulus was no pearls, no sugar, 180 PhP (3.70 USD), Tiger Sugar, fruit tea, without cream cheese, no ice with a total utility score of −1.489.

Pearl size was the most important attribute considered by the consumers with a score of 29.137%. Under the pearl size, big pearls were the most preferred while no pearls were the least preferred. On the other hand, brand and cream cheese inclusion were the least important attributes considered by the consumers with a score of 2.147% and 9.525%, respectively.

In line with the highest attribute, the most basic form of the milk and tea concoction consists of tea, milk, ice, and pearls sipped through a chunky straw to accommodate the large pearls [49]. These pearls are known for being the quintessential sinkers in milk teas and notable for their signature chewy (QQ) texture that makes the drink utterly distinctive and unique from the other drinks [50,51,52]. The inclusion of the pearls in milk tea was a novelty that quickly spread through the globe. It adds consistency and contributes to the fun factor of drinking milk tea rather than adding flavor [53,54,55]. These factors make milk tea drink exceptional. Having unique edible pearls in the milk tea makes the consumption experience stand out from other beverages [56,57]. Hence, milk tea is not complete without its pearls [57].

Milk tea is the crowd’s favorite beverage dessert mixed with an unbeatable topping, chewy boba pearls [9,58]. Thus, consumers prefer their milk tea with large pearls since it is the traditional topping and make the consumption experience exciting due to its addicting signature chewy texture and consistency. With the availability of smaller-sized pearls, consumers would choose it as an option for consumers having difficulty consuming a bigger-sized boba pearl. It could be supported with the result that the consumers would not prefer milk tea without any pearls having a result of −0.481 for the utility estimate.

Second, sugar level was found to be the second-highest attribute desired by the consumers (17.373%). It was also found that consumers prefer to have a normal sugar level in their beverages compared to more, less, and no sugar at all. These findings are supported by Tankeh [59] who stated that since diabetes is a major health problem, consumers partially learned to control their sugar intake by opting for normal sugar levels in their consumed beverages. Additionally, more sugar level was found to be the second preferred. Unsurprisingly, since respondents of this study are Filipinos, they will opt for this sugar level, as Lasco [60] stated that sugar is everywhere in Filipino cuisine, not just in desserts and savory dishes, but also in beverages that are rarely served without sugar (from coffee and tea to juices) and this has led to the conclusion that Filipinos have an exceptionally sweet tooth. Hence, Filipino consumers prefer a normal sugar level and enjoy the sweet flavor of the milk tea while controlling their sugar intake. Based on our result, consumers would opt to have more sugar compared to less (−0.05) and no sugar (−0.247) in their drink.

Third, ice was the third-highest attribute that impacted the decision of the consumers (17.190). The normal amount of ice was the most preferred, followed by more ice, less, and no ice. The significant difference between these levels indicates that due to the tropical climate, consumer would choose the level suited for the region’s weather [61,62,63]. The consumers desire their milk tea to be served as a cold beverage to quench their thirst on a hot day [64,65].

Fourth, type was considered as one of the attributes evaluated based on consumer preferences (13.421%). Milk tea was more liked by the consumers compared to fruit tea. These results are consistent a GMA News article [5], in which the majority of the consumers said they love milk tea due to its sweetness. For some consumers that prefer fruit tea, it was inferred that they tend to be conscious of drinking too many sweet beverages, which can negatively affect their health. Thus, they consider fruit tea as a healthier alternative to milk tea [65]. Based on the result, most consumers would still prefer milk tea as their choice rather than fruit tea.

Fifth, price was also an attribute considered based on the preferences of the consumer (11.207%). Among the levels, the cheapest price, 120 PhP (2.46 USD) was the most preferred, followed by 150 PhP (3.08 USD), and the expensive price, 180 PhP (3.70 USD) was the least preferred. The price of products or services is one of the concerned issues of consumers [66] and this is also applicable for the milk tea. De Jesus [16] concluded that the price list helped the consumers decide the milk tea they want if they see the price. Sangwai and Deshmukh [1] also stated that most consumers of milk tea are of younger generations, hence are sensitive about prices. In addition, Hashe [67] stated that most of the growth of the milk tea market focus on the young consumers. Han [68] conducted a study on the prices of milk tea in China and showed that most consumers are around 19–34 years old (88%). This supports the demographics considered in this study. Moreover, Han [68] stated that consumers are price conscious among any age group and gender. This therefore raises the market of milk tea across the world. In addition, Pangkey et al. [69] stated that the consumers would choose the milk tea shop that offers the most affordable price since most of them are students who are still receiving allowances from their parents. Similarly, as per Lee and Vega’s study [15], to achieve satisfaction and loyalty from consumers, the milk tea shops should offer affordable and reasonable prices for milk tea but should not compromise on quality. Hence, consumers want their milk teas to be cost-effective yet of premium quality.

Based on the results, brand and the cream cheese inclusion were least significant among the different attributes. Among the brands, GongCha was the most preferred brand, followed by Macao Imperial, CoCo, Chatime, and Tiger Sugar. GongCha is one of the brands that started the milk tea craze [70]. Its first store was established in 2011, making it the oldest brand from the five levels considered. GongCha is also known for their natural, healthy, and fresh drinks, prioritizing a healthier lifestyle for milk tea consumers [71]. Consumers are becoming more conscious of their health; thus, healthier options on milk tea are being considered when purchasing milk tea [15].

As seen in Figure 2 [43], Macao Imperial (13.9%), Chatime (11.4%), GongCha (10.2%), and CoCo (7.3%) were among the top 10 brands of milk tea. It was seen that Tiger Sugar (4.1%) ranked 9th among the 10 brands of milk tea [43]. From the results, Tiger Sugar was the least preferred brand of the consumers. Unlike other Brands, Tiger Sugar has only been in the milk tea industry for four years with only a few stores. Moreover, Tiger Sugar also has a fewer selection of milk tea compared to the other brands. These can be the factor as to why it was the least preferred by consumers.

With the consideration of cream cheese, consumers preferred milk tea with cream cheese rather than milk tea without cream cheese. Cream cheese adds a distinct texture and saltiness flavor to balance out the sweetness of milk tea [72]. Several studies have shown that the consumption of dairy food such as cream cheese may reduce the risk of obesity and cardiovascular disease [73,74]. Consumers prefer milk tea with cream cheese because of the additional flavor and health benefits it gives.

### 4.1. Contributions 

With the results of this study, consumers would tend to favor a drink that is cost-effective at the same time with a variation for their preferred drinks. The results of this study can help in developing a strategic marketing plan to survive the increasing competition in the beverage market, specifically in the milk tea market. Businesses under the milk tea industry should focus on prioritizing the choices and preferences of their consumers when it comes to the customization of their drinks. Moreover, having a variety of milk tea selections will help a business attract more consumers. For the beverage market in general, marketers should show the consumers how distinct their products are from other competitors. Since consumers give importance to the price and quality of a drink, marketers can highlight the benefits, affordability, and unique qualities of their beverages.

### 4.2. Practical Applications 

This study found that pearls and sugar levels were the two most important attributes affecting consumer preferences for milk tea. It indicates that the consumers desire the addition of pearls in milk tea as it has been usual for Asian countries to mix tapioca pearls in sweet beverages. The sweetness level of milk tea should be considered since consumers are becoming health conscious. Considering these findings, the researchers suggest that milk tea firms should focus on integrating these attributes in their product development and innovation to improve consumer satisfaction with milk tea drinks.

### 4.3. Limitations and Future Research

The significant contributions of this study must be seen in the light of some limitations. Starting off, the collection of data and preference measurement was done through an online survey during the COVID-19 pandemic. This resulted in a limited distribution of respondents, focusing on the age group of 16–24 year-olds since this age group is the most active online according to Vogels [75]. Furthermore, the researchers only focused on the preference of Filipino consumers for milk tea. Future research should gather more data from other countries, especially where the milk tea originated and are continuously increasing. This would help in comparing the preference of the general public towards milk tea. Lastly, the milk tea attributes that were examined in this study were based only on the most usual selection from popular brands in the Philippines. In future studies, it would be interesting to include more brands, new flavors, toppings, and sinkers (jelly, pudding, ice cream, or even cream puff). Hence, future research could further extend the findings regarding milk tea preference.

## 5. Conclusions

Milk tea has been one of the most popular purchased beverages globally since 2011 [76,77,78]. It is a milk and tea concoction composed of tea, milk, ice, and pearls shaken up together and slurped through chunky straws. This study integrated the Conjoint Analysis Approach using an orthogonal design in determining the most preferred combination of milk tea attributes of the consumers. A total of 1061 milk tea consumers voluntarily participated in the online survey that consisted of 34 combinations. Different attributes such as the size of tapioca pearls, sugar level, price range, brand, type of milk tea, the inclusion of cream cheese, and the amount of ice were evaluated.

The Conjoint Analysis revealed that pearl size was the most considered attribute affecting consumer preference. This was followed by sugar level, amount of ice, type of drink, price, the inclusion of cream cheese, and brand, which is determined as the least considered attribute by the consumers. This is the first and complete study that analyzes the consumer preference on milk tea attributes. The findings of this study will be useful for academicians [79,80] and even milk tea businesses in terms of the consumer preferences on different attributes of milk tea. Furthermore, the research may be extended to other countries’ milk tea brands and other types of beverages.

## Figures and Tables

**Table 1:** Descriptive statistics of the respondents (*n* = 1061).

Characteristics	Category	*n*	%
Gender	Male	274	25.8%
	Female	778	73.3%
	Other	9	0.8%
Age	Below 15	49	4.6%
	15–24	893	84.2%
	25–34	51	4.8%
	35–44	39	3.7%
	45–54	23	2.2%
	Above 54	6	0.6%
Monthly Salary/Allowance	<15,000 PHP	891	84.0%
	15,000–30,000 PHP	112	10.6%
	30,001–45,000 PHP	27	2.5%
	45,001–60,000 PHP	12	1.1%
	60,001–75,000 PHP	7	0.7%
	>75,000 PHP	12	1.1%
How many times in a week do you drink Milk Tea?	1	739	69.7%
	2	192	18.1%
	3	83	7.8%
	4	35	3.3%
	5	7	0.7%
	6	0	0
	Everyday	5	0.5%
Location	Region I	12	1.1%
	Region II	13	1.2%
	Region III	311	29.3%
	Region IV-A	228	21.5%
	Region IV-B	25	2.4%
	Region V	7	0.7%
	CAR	0	0
	NCR	390	36.8%
	Region VI	33	3.1%
	Region VII	24	2.3%
	Region VIII	5	0.5%
	Region IX	3	0.3%
	Region X	3	0.3%
	Region XI	1	0.1%
	Region XII	3	0.3%
	Region XIII	2	0.2%
	BARMM	1	0.1%

**Table 2:** Attributes of milk tea in the Philippines.

Attributes	Levels
Pearl size	Big pearls, Small pearls, No pearls
Sugar level	More sugar, Normal sugar, Less sugar, No sugar
Price	120 PhP (2.46 USD), 150 PhP (3.08 USD), 180 PhP (3.70 USD)
Brand	CoCo, Macao Imperial, GongCha, Tiger Sugar, Chatime
Type	Milk tea, Fruit tea
Cream cheese inclusion	with cream cheese, without cream cheese
Amount of ice	More ice, Normal ice, Less ice, No ice

**Table 3:** Stimulus.

Combination	Pearl Size	Sugar Level	Price	Brand	Type	Cream Cheese Inclusion	Amount of Ice
1	Small Pearls	No Sugar	150 PhP (3.08 USD)	Macao Imperial	Fruit Tea	with Cream Cheese	Less Ice
2	Big Pearls	Less Sugar	150 PhP (3.08 USD)	GongCha	Milk Tea	with Cream Cheese	No Ice
3	Small Pearls	Less Sugar	120 PhP (2.46 USD)	Macao Imperial	Milk Tea	without Cream Cheese	Less Ice
4	No Pearls	Less Sugar	180 PhP (3.70 USD)	CoCo	Fruit Tea	with Cream Cheese	Less Ice
5	No Pearls	No Sugar	120 PhP (2.46 USD)	GongCha	Milk Tea	without Cream Cheese	Less Ice
6	Big Pearls	No Sugar	150 PhP (3.08 USD)	Tiger Sugar	Fruit Tea	without Cream Cheese	Normal Ice
7	Small Pearls	Normal Sugar	150 PhP (3.08 USD)	CoCo	Milk Tea	without Cream Cheese	Normal Ice
8	Big Pearls	Less Sugar	180 PhP (3.70 USD)	GongCha	Fruit Tea	without Cream Cheese	Normal Ice
9	Big Pearls	Normal Sugar	150 PhP (3.08 USD)	GongCha	Milk Tea	with Cream Cheese	Less Ice
10	No Pearls	Less Sugar	150 PhP (3.08 USD)	CoCo	Milk Tea	without Cream Cheese	Less Ice
11	No Pearls	Normal Sugar	120 PhP (2.46 USD)	Tiger Sugar	Milk Tea	without Cream Cheese	No Ice
12	No Pearls	More Sugar	180 PhP (3.70 USD)	Macao Imperial	Milk Tea	without cream cheese	Normal Ice
13	No Pearls	More Sugar	150 PhP (3.08 USD)	Chatime	Fruit Tea	with Cream Cheese	No Ice
14	Big Pearls	More Sugar	120 PhP (2.46 USD)	CoCo	Fruit Tea	without Cream Cheese	Less Ice
15	Big Pearls	Less Sugar	120 PhP (2.46 USD)	Chatime	Milk Tea	with Cream Cheese	Normal Ice
16	Small Pearls	No Sugar	180 PhP (3.70 USD)	Chatime	Milk Tea	without Cream Cheese	More Ice
17	Small Pearls	More Sugar	120 PhP (2.46 USD)	GongCha	Fruit Tea	with Cream Cheese	Normal Ice
18	Big Pearls	Normal Sugar	120 PhP (2.46 USD)	Chatime	Fruit Tea	without Cream Cheese	Less Ice
19	Big Pearls	Normal Sugar	120 PhP (2.46 USD)	Macao Imperial	Milk Tea	with Cream Cheese	More Ice
20	Big Pearls	Less Sugar	180 pesos (3.70 USD)	Macao Imperial	Fruit Tea	without Cream Cheese	Normal Ice
21	Big Pearls	More Sugar	150 PhP (3.08 USD)	Macao Imperial	Fruit Tea	without Cream Cheese	More Ice
22	Big Pearls	No Sugar	120 PhP (2.46 USD)	CoCo	Fruit Tea	without Cream Cheese	No Ice
23	No Pearls	Normal Sugar	120 PhP (2.46 USD)	Macao Imperial	Fruit Tea	with Cream Cheese	Normal Ice
24	Big Pearls	No Sugar	120 PhP (2.46 USD)	CoCo	Milk Tea	with Cream Cheese	Normal Ice
25	Big Pearls	More Sugar	120 PhP (2.46 USD)	CoCo	Milk Tea	with Cream Cheese	More Ice
26	No Pearls	No Sugar	120 PhP (2.46 USD)	GongCha	Fruit Tea	with Cream Cheese	More Ice
27	Big Pearls	More Sugar	180 pesos (3.70 USD)	Tiger Sugar	Milk Tea	with Cream Cheese	Less Ice
28	Small Pearls	No Sugar	150 PhP (3.08 USD)	CoCo	Milk Tea	without Cream Cheese	Less Ice
29	Big Pearls	Normal Sugar	180 pesos (3.70 USD)	GongCha	Fruit Tea	without Cream Cheese	More Ice
30	Big Pearls	Less Sugar	120 PhP (2.46 USD)	Macao Imperial	Fruit Tea	without Cream Cheese	No Ice
31	Small Pearls	More Sugar	120 PhP (2.46 USD)	GongCha	Milk Tea	without Cream Cheese	No Ice
32	Small Pearls	Normal Sugar	180 PhP (3.70 USD)	CoCo	Fruit Tea	with Cream Cheese	No Ice
33	Small Pearls	Less Sugar	120 PhP (2.46 USD)	Tiger Sugar	Fruit Tea	with Cream Cheese	More Ice
34	Big Pearls	No Sugar	180 PhP (3.70 USD)	Macao Imperial	Milk Tea	with Cream Cheese	No Ice

**Table 4:** Utilities.

Attributes	Preference	Utility Estimates	Std. Error
	Big Pearls	0.330	0.045
Pearl Size	Small Pearls	0.151	0.053
	No Pearls	−0.481	0.053
	More Sugar	0.060	0.059
Sugar Level	Normal Sugar	0.237	0.059
	Less Sugar	−0.050	0.059
	No Sugar	−0.247	0.059
	120 PhP (2.46 USD)	0.165	0.045
Price	150 PhP (3.08 USD)	−0.018	0.053
	180 PhP (3.70 USD)	−0.147	0.053
	CoCo	0.007	0.063
	Macao Imperial	0.010	0.063
Brand	GongCha	0.018	0.063
	Tiger Sugar	−0.042	0.082
	Chatime	0.006	0.082
Type	Milk Tea	0.187	0.034
	Fruit Tea	−0.187	0.034
Cream Cheese	With Cream Cheese	0.133	0.034
Inclusion	Without Cream Cheese	−0.133	0.034
	More ice	0.065	0.059
Amount of Ice	Normal ice	0.227	0.059
	Less ice	−0.040	0.059
	No ice	−0.252	0.059
(Constant)		4.058	0.039

**Table 5:** Averaged importance score.

Importance Values	Score
Pearl size	29.137
Sugar level	17.373
Price	11.207
Brand	2.147
Type	13.421
Cream cheese inclusion	9.525
Ice	17.190

**Table 6:** Stimulus rank.

Combination	Pearl Size	Sugar Level	Price	Brand	Type	Cream Cheese Inclusion	Amount of Ice	Total	Rank
1	Small Pearls	No Sugar	150 PhP (3.08 USD)	Macao Imperial	Fruit Tea	with Cream Cheese	Less Ice	−0.198	27
2	Big Pearls	Less Sugar	150 PhP (3.08 USD)	GongCha	Milk Tea	with Cream Cheese	No Ice	0.348	12
3	Small Pearls	Less Sugar	120 PhP (2.46 USD)	Macao Imperial	Milk Tea	without Cream Cheese	Less Ice	0.29	15
4	No Pearls	Less Sugar	180 PhP (3.70 USD)	CoCo	Fruit Tea	with Cream Cheese	Less Ice	−0.471	31
5	No Pearls	No Sugar	120 PhP (2.46 USD)	GongCha	Milk Tea	without Cream Cheese	Less Ice	−0.531	32
6	Big Pearls	No Sugar	150 PhP (3.08 USD)	Tiger Sugar	Fruit Tea	without Cream Cheese	Normal Ice	−0.07	24
7	Small Pearls	Normal Sugar	150 PhP (3.08 USD)	CoCo	Milk Tea	without Cream Cheese	Normal Ice	0.658	7
8	Big Pearls	Less Sugar	180 PhP (3.70 USD)	GongCha	Fruit Tea	without Cream Cheese	Normal Ice	0.352	11
9	Big Pearls	Normal Sugar	150 PhP (3.08 USD)	GongCha	Milk Tea	with Cream Cheese	Less Ice	0.847	4
10	No Pearls	Less Sugar	150 PhP (3.08 USD)	CoCo	Milk Tea	without Cream Cheese	Less Ice	−0.423	30
11	No Pearls	Normal Sugar	120 PhP (2.46 USD)	Tiger Sugar	Milk Tea	without Cream Cheese	No Ice	−0.319	29
12	No Pearls	More Sugar	180 PhP (3.70 USD)	Macao Imperial	Milk Tea	without Cream Cheese	Normal Ice	0.017	23
13	No Pearls	More Sugar	150 PhP (3.08 USD)	Chatime	Fruit Tea	with Cream Cheese	No Ice	−0.739	34
14	Big Pearls	More Sugar	120 PhP (2.46 USD)	CoCo	Fruit Tea	without Cream Cheese	Less Ice	0.202	18
15	Big Pearls	Less Sugar	120 PhP (2.46 USD)	Chatime	Milk Tea	with Cream Cheese	Normal Ice	0.998	2
16	Small Pearls	No Sugar	180 PhP (3.70 USD)	Chatime	Milk Tea	without Cream Cheese	More Ice	0.176	20
17	Small Pearls	More Sugar	120 PhP (2.46 USD)	GongCha	Fruit Tea	with Cream Cheese	Normal Ice	0.567	8
18	Big Pearls	Normal Sugar	120 PhP (2.46 USD)	Chatime	Fruit Tea	without Cream Cheese	Less Ice	0.378	10
19	Big Pearls	Normal Sugar	120 PhP (2.46 USD)	Macao Imperial	Milk Tea	with Cream Cheese	More Ice	1.127	1
20	Big Pearls	Less Sugar	180 PhP	Macao Imperial	Fruit Tea	without Cream Cheese	Normal Ice	0.344	13
			(3.70 USD)						
21	Big Pearls	More Sugar	150 PhP (3.08 USD)	Macao Imperial	Fruit Tea	without Cream Cheese	More Ice	0.127	21
22	Big Pearls	No Sugar	120 PhP (2.46 USD)	CoCo	Fruit Tea	without Cream Cheese	No Ice	−0.317	28
23	No Pearls	Normal Sugar	120 PhP (2.46 USD)	Macao Imperial	Fruit Tea	with Cream Cheese	Normal Ice	0.104	22
24	Big Pearls	No Sugar	120 PhP (2.46 USD)	CoCo	Milk Tea	with Cream Cheese	Normal Ice	0.802	5
25	Big Pearls	More Sugar	120 PhP (2.46 USD)	CoCo	Milk Tea	with Cream Cheese	More Ice	0.947	3
26	No Pearls	No Sugar	120 PhP (2.46 USD)	GongCha	Fruit Tea	with Cream Cheese	More Ice	−0.534	33
27	Big Pearls	More Sugar	180 PhP (3.70 USD)	Tiger Sugar	Milk Tea	with Cream Cheese	Less Ice	0.775	6
28	Small Pearls	No Sugar	150 PhP (3.08 USD)	CoCo	Milk Tea	without Cream Cheese	Less Ice	−0.093	25
29	Big Pearls	Normal Sugar	180 PhP (3.70 USD)	GongCha	Fruit Tea	without Cream Cheese	More Ice	0.477	9
30	Big Pearls	Less Sugar	120 PhP (2.46 USD)	Macao Imperial	Fruit Tea	without Cream Cheese	No Ice	−0.117	26
31	Small Pearls	More Sugar	120 PhP (2.46 USD)	GongCha	Milk Tea	without Cream Cheese	No Ice	0.196	19
32	Small Pearls	Normal Sugar	180 PhP (3.70 USD)	CoCo	Fruit Tea	with Cream Cheese	No Ice	0.236	16
33	Small Pearls	Less Sugar	120 PhP (2.46 USD)	Tiger Sugar	Fruit Tea	with Cream Cheese	More Ice	0.235	17
34	Big Pearls	No Sugar	180 PhP (3.70 USD)	Macao Imperial	Milk Tea	with Cream Cheese	No Ice	0.308	14

**Table 7:** Correlation.

	Value	Significance
Pearson’s R	0.966	0.000
Kendall’s Tau	0.861	0.000
Kendall’s Tau for Holdouts	1.000	

## Data Availability

The data presented in this study are available on request from the corresponding author.
